# “You want to get on with the rest of your life”: a qualitative study of health-related quality of life in gout

**DOI:** 10.1007/s10067-015-3039-2

**Published:** 2015-08-06

**Authors:** Priyanka Chandratre, Christian D. Mallen, Edward Roddy, Jennifer Liddle, Jane Richardson

**Affiliations:** Research Institute of Primary Care and Health Sciences, Keele University, Keele, Staffordshire ST5 5BG UK

**Keywords:** Focus group interviews, Gout, Health-related quality of life, Primary care, Qualitative study, Thematic analysis

## Abstract

The objective of the study is to examine the impact of gout and its treatments on health-related quality of life (HRQOL) using focus group interviews. From the baseline phase of a cohort study of HRQOL in gout, 17 participants (15 males, mean age 71 years) with varying attack frequency and treatment with and without allopurinol participated in one of four focus group interviews. All interviews were audio-recorded and transcribed verbatim. Data was analysed thematically. Physical and psychosocial HRQOL in gout was affected by characteristics of acute gout (particularly the unpredictable nature of attacks, location of joint involved in an attack, pain and modifications in lifestyle), lack of understanding of gout by others (association with unhealthy lifestyle, symptoms ridiculed as non-severe and non-serious) as well as participants (not considered a disease) and the lack of information provided by physicians (about causes and pharmacological as well as non-pharmacological treatments of gout). Participants emphasised the impact of acute attacks of gout and prioritised dietary modifications and treatment of acute attacks over long-term urate-lowering therapy. Characteristics of acute gout, lack of understanding and information about gout and its treatments perpetuate poor HRQOL. HRQOL (maintenance of usual diet and reduced frequency of attacks) was associated with urate-lowering treatment. Better patient, public and practitioner education about gout being a chronic condition associated with co-morbidities and poor HRQOL may improve understanding and long-term treatment of gout.

## Introduction

Gout is the commonest inflammatory arthritis, affecting 2.5 % of the UK population [1] and causes attacks of acute gouty arthritis, joint damage and chronic pain. It is associated with co-morbidities (obesity, hypertension, diabetes, ischaemic heart disease, chronic kidney disease and treatment with diuretics) [2, 3] and socio-demographic features (older age, male gender, ethnicity and lower socio-economic status) [4–6]. Given the complex links between gout, co-morbidities and socio-demographic characteristics, health-related quality of life (HRQOL) in gout is likely to be associated with all these patient characteristics. HRQOL is defined as: *“The value assigned to the duration of life as modified by impairments, functional states, perceptions and social opportunities that are influenced by disease, injury, treatment or policy”* [7].

To date, qualitative studies of gout have explored HRQOL but have focused on the knowledge and beliefs of patients and providers and perceived barriers to effective treatment rather than on the impact of gout and its treatment on quality of life [8–13]. These studies were conducted using one-to-one interviews with patients [10, 12] in mixed primary and secondary care settings [9]. One secondary care-based study used nominal group interviews to assess the impact of gout on HRQOL but focused on the influence of gender and race on HRQOL [8]. To our knowledge, this is the first primary care-based qualitative study to explore patients’ perspectives on how gout and its treatments affect HRQOL, using focus group interviews.

## Methods

Participants for this study were recruited from the baseline phase of a primary care-based cohort study of HRQOL in gout [14]. A sub-sample of 120 baseline responders with a primary care Read code diagnosis of gout or prescription of colchicine or allopurinol in the preceding 2 years were invited to participate in focus group interviews. Drawing on the expertise of the research team, a purposive sampling framework was developed to include participants whose experiences encompassed a range of frequency of attacks over the last 12 months and a proportion taking allopurinol. Since febuxostat is used infrequently in UK primary care, interview participants were not purposively sampled according to febuxostat use.

**Fig. 1:**
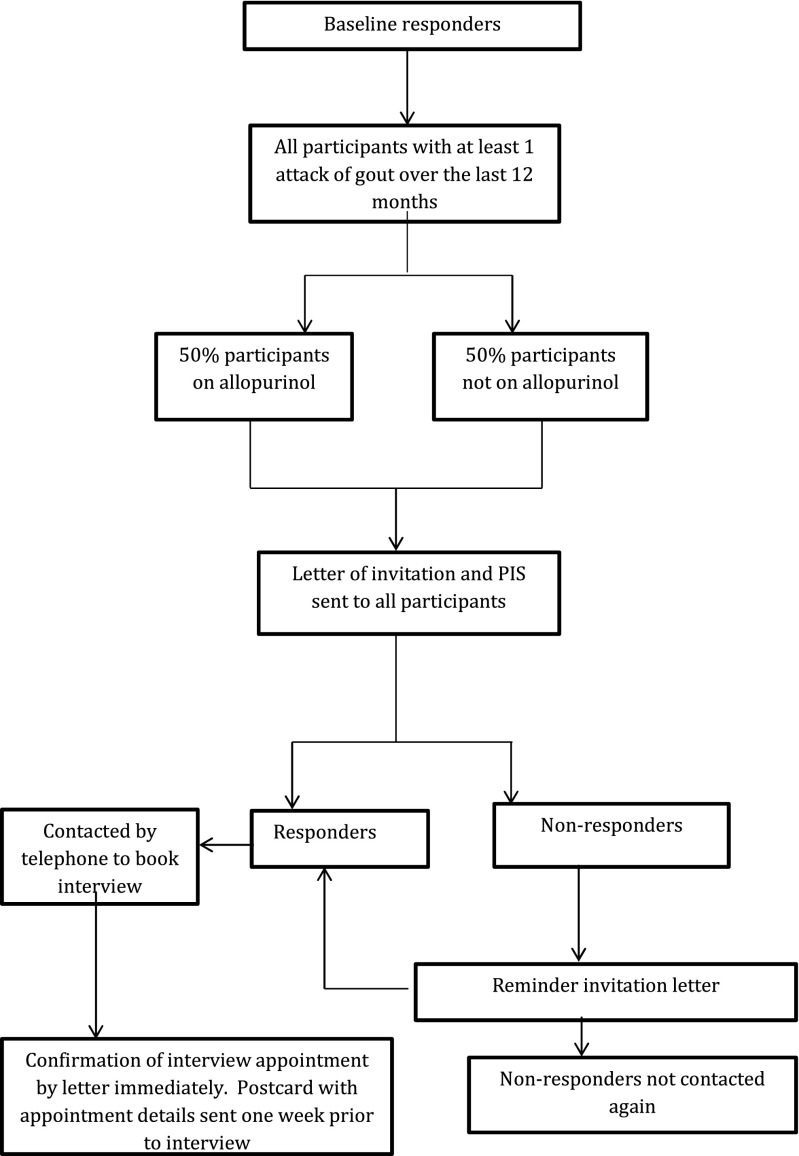
Recruitment process for the qualitative focus group

Non-responders were sent a reminder invitation letter after 2 weeks: Those who did not reply to the reminder letter were not contacted again. Forty-two potential participants who agreed to take part in the interviews were telephoned by the researcher to arrange an interview. Nineteen of these 42 confirmed ability to attend one of the four allocated interview dates. Two did not attend due to unforeseen circumstances, leaving 17 participants. Each of the four focus groups had between three and five participants. Details of the recruitment process are illustrated in Fig. 1.

Prior to commencing the interview, the procedures outlined in the participant information sheet were discussed with each participant. Participants were given the opportunity to ask questions. Written informed consent to take part in the study (including the use of quotations) was obtained from all participants and confirmed at the end of the interview. Three group interviews were held at Keele University and one at a general practice. Ethical approval was gained from the North West Liverpool East Local Research Ethics Committee (REC reference number 12/NW/0297).

Focus groups rely on the interaction between group participants to generate novel ideas and promote discussion around the emerging topics [15]. The moderator (PC) used a topic guide to guide the interview, which had been developed in conjunction with five expert patients with gout from the research centre’s Research Users Group. The topic guide was pre-determined but not prescriptive, and participants were encouraged to lead the discussion. The main question of the focus group enquiry was: “What impact has gout and its treatment had on your Quality of Life?” All interviews were audio-recorded and transcribed verbatim.

### Thematic analysis

Thematic analysis was based on Braun and Clarke’s framework [16], modified by combining reviewing and defining themes into one stage:

I. Familiarisation with the data set
II. Generating and clustering codes together
III. Identification of themes
IV. Review and definition of themes
V. Production of the report

The original transcripts were scrutinised by three researchers (PC, JR, JL) for data relevant to the impact of gout (and its treatments) on all aspects of quality of life, which were then coded. Codes identified by the three researchers were largely similar, and any differences were discussed until a consensus was reached. Codes used to annotate the main text that were similar in nature were clustered together into themes. Similar themes were organised under one overarching theme or higher order descriptive label. Thematic analysis was data driven (inductive) as far as possible; however, previous clinical experience may inevitably have contributed to some degree of deductive analysis. Data analysis and interpretation were iterative as new themes developed on repeated readings of the transcripts, until no new themes could be identified (theoretical saturation) [10].

## Results

Seventeen people participated in the interviews (mean age 71 years, 15 males). One participant accompanied another participant with gout (for whom she was a carer) but did not have gout herself. Participant characteristics are presented in Table 1.

**Table 1:** Participant characteristics

Gender	Age (years)	Interview location GP/Keele	Number of attacks in last 12 months	Taking allopurinol
F	76	GP	5	Yes
M	75	Keele	5	No
M	73	GP	2	No
M	55	Keele	3	Yes
M	67	Keele	1	No
M	68	Keele	3	Yes
M	85	Keele	3	No
M	77	Keele	2	Yes
M	72	GP	2	Yes
M	81	Keele	3	Yes
M	68	Keele	2	Yes
M	64	Keele	2	No
M	64	Keele	2	Yes
M	78	Keele	3	Yes
M	60	Keele	4	No
M	63	Keele	3	Yes
M	75	Keele	5	No
F	Unknown	Keele	NA	NA

*M* male, *F* female, *GP* general practitioner, *NA* not applicable

Three overarching themes were developed: characteristics of gout, understanding of gout and beliefs about treatment of gout amongst the participants. The impact of gout on HRQOL is outlined through these themes and sub-themes presented below, illustrated with relevant quotations from the transcripts (Tables 2, 3 and 4).

**Table 2:** Participant quotations to illustrate the impact of gout characteristics on HRQOL

Higher order theme	Sub-theme	Quotations within transcripts
Gout characteristics	The impact of gout attacks	I mean a toe is relatively innocuous, if you’ve got it in your knees or hips or something, then yeah, it’s a little more worrying
		So I can’t really go anywhere or do anything in that sense
		You’re so bored sat there not being able to move your foot, [laughter] that you get psychological side effects.
		I’ll get into freezing cold water and sit there. [yeah] I take that pain to take that off
		You can’t turn over, when you’re half asleep, you accidently touch something. You’re frightened that she’s going to touch it
		But mine lies all over my body, everywhere. From one to another. [right] All down one side, well everywhere
		You really bang your head against the wall
		It gets that painful I’ll cry. I can’t get rid of it.
		If it breaks, [yeah] you go to the hospital, put it in plaster, and you’re—a bit of a throbbing and it’s gone, but with gout it’s bang, bang, bang for days and days
	Unpredictable nature of attacks	The only reason that erm I went back this time to—to see about it was the fact that I was a little bit frightened if I was going to go on holiday the next day it was going to clobber me that day
		It’s the unpredictability of it, you know, you make a plan to, I don’t know, maybe go to theatre in five weeks’ time and when it gets closer you think god, I hope I don’t get gout just the night before
	Lifestyle modification	Well I couldn’t get my shoe on, last—a week ago since my last one
		When I found out it was gout I changed my lifestyle and stopped drinking
		I have cherries. And I have seeds sometimes, celery seeds
		Because the damp weather, the cold and damp weather, is just not helping him at all. And they moved, they sold up and they moved to warmer climates
		I stopped doing these high impact erm exercises, I stopped long distance walking, because it was painful
		Like it’s office work now, like you know a desk job now
		Well we can’t go out and do the same things
		I could go out and leave him. [right, yeah] But there’s no way I would. [okay] So it does have an effect on the whole unit
		I’m a long distance runner, so when I can’t run like I hate it.

**Table 3:** Participant quotations to illustrate the impact of understanding of gout on HRQOL

Higher order theme	Sub-theme	Quotations within transcripts
Understanding of gout	Over-indulgence and dietary modifications	Yeah I know I kind of guess when I might be getting one, [yeah] by the fact that I’ve over indulged somewhere.
		Oh they put everything on there. What am I going to eat? You have to take it with a pinch of salt.
		When I looked onto NHS Direct, after I’d got it, that frightens the life out of you if you do anything because you get five pages
		It’s just a great muddle about when it comes to food
		Give them a 12-month diary or something like that. [right] And write each day what they’ve done that day. [okay] What they’ve drunk that day. What they’ve eaten that day. [yeah] And do a research programme like that and maybe you could come up with some facts
	Gout not a disease but natural	For me, disease is something like malaria and erm… But it isn’t is it, it’s just a build-up of stuff that’s naturally in your body
		I suppose I was a bit in self-denial, I don’t suffer from gout
		There’s more people than what we think who get it a bit, not coming forward and saying this is a bigger serious problem
		In fact I would put it down to aches and pains getting aged really rather than anything
	Gout considered humorous by others and only understood by close family and friends	I think there’s certain diseases that are quite humorous to—and they’re not, but they’re humorous to everybody else who hasn’t got them
		You don’t brag about it do you?
		Straightaway it’s with the well-off people and [that’s right, yeah] and the rich food.
		It’s this thing erm…they don’t realise what it is and they just use the old wives’ tale, the port and pheasant, rich living
		It happens so quick, people just don’t believe it.
		When you’ve got gout your partner or friend or whatever, if they see you with gout when it’s bad, they suddenly realise how bad it is
		I don’t think it’s perceived to be life threatening, whereas cancer and heart attacks are
		They should spend more money on stuff which we ain’t brought this on ourselves, [yeah] it’s because it’s an illness, it’s—whatever it is, we’ve got with us, whereas drugs and—they’ll spend money
	Lack of information from health care practitioner	I found out for myself basically. [okay] So the doctor didn’t really explain it that well
		We’ve all got ignorance of it. Doctors don’t sort of explain exactly what it is
		I’d like to know the side effects though, properly [yeah] from a doctor, and not from the internet
		No, you go in, you go in, you’re the doctor, how much do you drink? I said I don’t drink doctor. But as I say it’s still treated as a bit of a thing, you know. I think doctors do actually. You know, you’ve been drinking. How much do you drink?
		“I would say my GP almost dismissed my view that [yeah] the attacks were brought on when I stressed the joint, but on the NHS site, definitely it states [yeah] that if you stress a joint it can instigate the gout”
		“But you couldn’t talk to my doctor about it, he wasn’t interested”

**Table 4:** Participant quotations to illustrate the impact of treatments on HRQOL

Higher order theme	Sub-theme	Quotations within transcripts
	Lack of contact with HCP	A female participant reported that her general practitioner (GP) was reluctant to refer her to a specialist even when her gout was sub-optimally controlled
Treatment		Have a supply if I can feel it coming on, because I’ve got a spare box at home
		I dropped it down myself to one a day, I don’t know what the doctor will say when I tell him
		So it takes three, like it can take five days to see my doctor. You know, so by the time I get in there it’ll probably have eased down a lot
	Reluctance to prescribe and take allopurinol	He says I wouldn’t really recommend it if you can get away with it, just come in if you start getting an attack
		I find it quite manageable with anti-inflammatory tablets I take for it
		I said I’m not being funny here but can I have this one please because this one seems to be the new one, and much better. She didn’t offer it because it’s obviously more expensive
		I’m old enough now that another tablet for the rest of my life doesn’t make a lot of difference
		I find mine just goes quickly, so I’m tremendously happy, I wouldn’t want to be on long term Allopurinol, not because there’s anything wrong with it, or anything, or anything else, I’m very, very content with what I’ve got
	Concerns about side effects of treatment	Because of the other medication that he takes, the gout tablets don’t sit well
		My kidney function, he always checks because I think it’s on the border line, so I think that might have been one of the reasons he was a little bit wary about erm prescribing Allopurinol
		And then you go—and then you get gout, it gives you gout.
		My medic said that Allopurinol can actually cause gout to flare up again. If I had any problems, any pain, [yeah] to stop taking it immediately.
	Benefits of treatment	You go two for I think it’s two months, I’ve forgotten now, [yes] and then you go to three, and then that is—that’s a miracle
		Go to the doctors and get the pills… I wish he’d done it two years ago
		“Well I’m still eating mussels and king prawns and everything like that. The Allopurinol I suppose is to let you do that isn’t it?”

### Characteristics of gout

#### The physical and psychological impact of gout attacks

Participants reported pain affecting multiple sites in the body and varying in intensity (see Table 2). The duration of severe pain could last as long as 3 weeks, but some participants reported a low intensity of pain lasting almost a decade. Desperation to relieve the severe pain of gout was also apparent from some of the extreme self-management techniques). Anticipated worsening of pain upon contact of the affected body part with another person or object in bed reduced comfort and the quality of sleep. Gout was perceived to be more severe if pain was located in larger joints during an attack compared to smaller joints (Table 2) and considered more painful than a fractured bone. Gout caused isolation through reduced mobility arising from pain and swelling in the joints. Being immobile, housebound and unable to do things led to feelings of boredom.

#### Unpredictable nature of attacks

The unpredictable onset of acute attacks led to difficulties in planning activities or social engagements in the future, illustrating the direct impact of gout on HRQOL in terms of social opportunities. Some participants were reluctant to make commitments which might not be fulfilled in the event of a sudden attack. Fear of recurrent and unpredictable attacks of gout led one participant to start treatment with allopurinol, which he would not have considered otherwise (Table 2).

#### Modification of environment and lifestyle

Symptoms of gout caused hindrance in performing activities of daily living which meant that participants made modifications in their lifestyle, place of living and work environment. Gout not only limited the lifestyles and hence HRQOL of participants affected by it but also of the family member (carer) who reported feeling unhappy or guilty enjoying activities without the person with gout (Table 2).

### Understanding of gout

#### Over-indulgence as a cause for gout and dietary modifications

Although some participants considered dietary modifications a key to preventing recurrent attacks (particularly if they thought their previous dietary habits had been ‘overindulgent’), others reported uncertainty regarding diet and its role in causing and treating gout. There was a lack of information from ‘trusted’ sources such as health care practitioners, and participants relied upon both National Health Service (NHS) endorsed and unendorsed websites for detailed dietary information. Self-discovered sources of information were considered to be overwhelming and frightening (Table 3). Participants also reported lack of enjoyment of previously enjoyed activities associated with extremely restricted dietary lifestyles. This aspect of HRQOL is an important one and, at the same time, a confusing one for people with gout.

#### Gout not a disease

The stigma associated with stereotypical ideas of gout affecting those who led an unhealthy lifestyle (high consumption of meat and alcohol) was still widely prevalent in society according to participants. However, some participants did not consider personal actions such as lifestyle choices to be a key cause of gout as they saw it as an illness resulting from a ‘natural’ accumulation of metabolites in the body rather than a disease. Often, symptoms of gout (joint aches and pains) were attributed by participants to part of a ‘normal’ ageing process, leading to dismissal of the diagnosis.

#### Gout considered humorous and only understood by close contacts

Participants considered gout to be a serious and extremely painful condition and were angered by others not taking it seriously. Participants felt that the rapid onset of symptoms, and others’ understanding of it as a non-fatal condition, meant that there was disbelief of the severity of symptoms and the condition. Perceived lower priority for research into gout (compared to other conditions such as drug misuse or dependency which were viewed as self-inflicted) was considered synonymous with the lower severity assigned to it by health care practitioners (Table 3). Awareness and understanding of gout were considered better amongst family and friends who realised the severity of symptoms after being involved in the care or observation of someone who had gout. The perceptions of other people can be seen to have an impact on patients’ HRQOL.

#### Lack of information from health care practitioners

Lack of information prompted participants to conduct their own internet searches on causes and treatments of gout. They were, however, concerned about the quality and authenticity of information available via these internet sources. Participants felt that health care practitioners assumed that their gout was due to excessive consumption of alcohol (Table 3), leading to a reported lack of rapport between health care practitioners and patients.

### Beliefs about treatment of gout

#### Lack of contact with health care practitioner

Self-treatment of acute attacks of gout with non-pharmacological methods was reported by participants. Obtaining topical or oral non-steroidal anti-inflammatory drugs (NSAIDs) from the pharmacy was preferred by some participants to presentation to the general pactitioner (GP) for treatment. Some confessed to treating recurrent attacks with left over NSAIDs (obtained originally from a previous consultation). Participants complained of the unavailability of appointments at their GP surgery and the spontaneous resolution of symptoms by the time they were seen. Lack of presentation to the GP for recurrent attacks may lead to lack of opportunity to understand and address the long-term consequences of chronic progressive gout and its associated co-morbidities on HRQOL for the patient.

#### Reluctance to prescribe and take allopurinol

A recurrent theme in the interviews was that lifelong urate-lowering therapy (ULT) treatment with allopurinol was not widely advocated by health care practitioners if the patients had single or infrequent attacks or in the presence of co-existing renal impairment. Instead, treatment of acute attacks only with NSAIDs was often reportedly advised by health care practitioners, as well as being the preferred approach for some participants (see Table 4). Those who had mild symptoms were content without any treatment at all or quick resolution of symptoms with NSAIDs. Reluctance to take lifelong treatment (allopurinol) was expressed by a few participants despite having no particular concerns regarding allopurinol. These participants may consider taking lifelong medication a burden. Some participants reported being less concerned about taking allopurinol for the remainder of their lives as they grew older (Table 4). Not taking treatment can have a negative effect on HRQOL.

#### Concerns about side effects of treatment

Lack of information about the possibility of an acute attack due to allopurinol initiation or titration caused concerns for some participants. Other participants were informed of this possibility but were incorrectly advised to discontinue treatment with allopurinol should an acute attack occur. Some participants (including the carer) were worried about interaction between allopurinol and other medications taken for co-morbid conditions. Treatment of gout with allopurinol was considerably harder in the presence of other co-morbid conditions such as renal disease, according to some participants.

#### Perceived benefits of treatment

Some participants wished for earlier treatment with allopurinol once they realised that treatment could reduce the frequency of attacks (Table 4). Treatment with allopurinol was perceived to improve HRQOL by reducing the frequency of recurrent attacks.

## Discussion

The impact of gout and its treatments on broad physical, social functioning and mental health [17] components of HRQOL was represented through three higher order themes: gout characteristics, understanding of gout and treatments for gout. The effect on physical HRQOL was evident through its characteristic symptoms of pain and swelling in the affected joint, leading to reduced mobility and potential adverse effect on psychological HRQOL. Social HRQOL may be affected by the unpredictable nature of attacks and modifications in lifestyle. Participants’ treatment preferences and lack of knowledge about the benefits of ULT may contribute towards poor HRQOL in gout.

The impact of gout symptoms on physical functioning and psychological HRQOL [8, 10], work absence and productivity has been described previously [18]. Under-reporting of gout due to reluctance in accepting the diagnosis (stigma attached with the stereotypical phenotype of those who get gout) and stoicism due to societal perceptions (non-serious) have also been found previously [10]. Non-presentation to a health care practitioner for treatment of further attacks prevents the opportunity to discuss the association of gout with permanent joint damage, disability and co-morbidities [19] and may lead to poor HRQOL, which can be addressed through treatment with a urate-lowering agent such as allopurinol. A previous observational cohort study has shown statistically and clinically meaningful improvement in HRQOL (through reduction in serum uric acid (SUA) and the frequency of attacks) in participants with chronic gout treated with ULT [ 20]. Participants in our study highlighted lack of awareness of the need for lifelong ULT, concerns about side effects, induction of acute attacks with ULT, concerns regarding polypharmacy causing adverse drug interactions and perception that treatment is only needed for acute attacks as reasons for not taking ULT, which have been common to other qualitative studies using semi-structured or nominal group interviews [9, 12, 13]. Such beliefs may contribute towards under-utilisation of ULT in primary care [21]. Leaving recurrent attacks untreated may lead to progressive gout which has been previously associated with negative experiences [10]. Co-morbidities such as renal impairment have been independently associated with poor HRQOL [22]. Better psychological HRQOL (measured by the Short Form 36 scale) in adults >70 years of age with treatment failure gout compared to younger subjects and general population has been seen previously [23].

One important concept identified in this study is the distinction between gout as an illness (social meaning of the condition) rather than a disease (a biological condition) [24]. This belief may be rooted within social constructionism (illnesses are socially constructed at an experiential level which is based upon the individual’s understanding of the disease and perceptions of his or her identity post diagnosis) [25]. Another addition to the findings of existing studies is that participants in this study considered the unpredictable nature of attacks and location of joint pain and swelling to be of utmost importance in affecting their HRQOL. These findings may explain how previously noted features of quantitative studies [26, 27] such as attack frequency and number of joints involved during an attack affect HRQOL. Although well-recognised as features of gout by health care practitioners, associated co-morbidities [28] and tophi were noticeably not discussed amongst participants of this study, which may imply that they did not consider these to affect HRQOL. Although some participants acknowledged that treatment of gout was difficult in the context of co‐morbidities such as renal diseases, none attributed co‐morbid conditions as a risk factor for hyperuricaemia and gout. Untreated co-morbid conditions in gout may impair HRQOL independent of gout factors [22]. Tophi have also been associated with poor HRQOL (measured by generic questionnaires) in other studies [27, 29]. This may be due to the fact that tophi are less common in primary care, where our sample was taken from, compared to secondary care, where much previous research has been conducted.

Other findings of our study supported by existing literature include the desire for greater health care practitioner-led information [9] and the emphasis on diet as a key causative factor in development of gout [13, 30]. However, whereas participants in our study were keen on modifying their lifestyle to prevent recurrent attacks, participants in the other study [30] did not perceive gout to be influenced by their personal actions. Such differences in findings may be attributed to the sampling frame (participants with disease duration <10 years), geographical location (New Zealand) and study design (cohort study conducted using the Brief Illness Perception Questionnaire).

### Strengths of the study

To our knowledge, this is the first focus group study to evaluate the effects of gout and its treatment on HRQOL. Focus group interaction between participants may have contributed towards uninhibited discussion [31]. Group interaction also promoted exchange of ideas, anecdotes and information, which may have led to novel themes that would not have developed in a one-to-one setting. Such themes add depth to our understanding of the impact of gout. Although initially designed to study the impact of gout and its treatment on HRQOL, the group interactions went beyond these realms and introduced discussions about beliefs and knowledge of gout, both of which link into the impact on HRQOL. The patient sample was broadly representative of the experience of the primary care population with gout in the UK as it covered a range of attack frequencies and just over half of the participants were on ULT (allopurinol). Participation of a carer for someone with gout added a first-hand perspective of the impact of gout on family and friends. Independent reviews of the transcripts by three researchers added robustness to the identification of the codes, ensuring that they reflected participants’ views as closely as possible.

### Limitations of the study

Limitations of this study include a sample consisting of exclusively Caucasian and mostly male participants, reducing the applicability of the findings beyond these ethnic and gender groups. However, data from qualitative research is useful in enhancing understanding of the social phenomenon through volume, depth and complexity [31]. Previous qualitative studies have examined the impact of gender and race on HRQOL in people with gout but have not explored the influence of age [8, 13]. Owing to the study design and sample, we were unable to examine this aspect of HRQOL in gout which should be the focus of future research studies. Although largely present in a passive role, the presence of a moderator and research assistant may have influenced the participant responses.

### Implications for clinical practice and further research

From the participants’ perspective, HRQOL is very much influenced by the acute symptoms and ‘unpredictable’ nature of gout. However, patients need to be made aware of the irreversible joint damage (regardless of joint location and size) associated with untreated chronic gout [32] which may lead to chronic poor HRQOL over time. At present, there appears to be patient preference for short-term symptomatic treatment of gout over ULT. Health care practitioners need to be aware and be able to explain to patients that ULT is necessary to see a long-term reduction in SUA thereby reducing the frequency of attacks, shrinkage and dissolution of tophi [33], leading to an improvement in HRQOL long term [20]. As well as the possibility of onset of an acute attack of gout, they should be advised about its management and the need to continue allopurinol through the duration of the acute attack.

Patients also need to be made aware by health care physicians that gout is a recognised independent risk factor for co-morbidities [34–36] which should be screened for and treated alongside gout as they are known to be independently associated with poor HRQOL [22]. Greater awareness of gout in the context of co-morbidities and genetic susceptibility [37] may steer away from the historical association of gout with excessive intake of alcohol and food [2, 32]. Greater information on the role of diet and exercise from health care practitioners could also promote lifestyle modification and patient engagement in management of the condition.

The influence of gender (females) and cultural perceptions or beliefs (different ethnicities) on HRQOL could also be explored through further qualitative studies. Although lack of information from clinicians was a recurrent theme, this was the participants’ perception and it may be useful to explore clinicians’ perspective on this issue as well as their perceptions of what affects HRQOL in gout.

#### Key messages

- HRQOL was impaired due to gout characteristics, lack of understanding and information about gout.
- Patients expressed a general preference for treatment of acute attacks of gout over lifelong ULT.
- Better patient (and physician) education is warranted to address these factors which perpetuate poor HRQOL.
